# Fundamental movement skills in preschoolers before and during the COVID-19 pandemic in Japan: a serial cross-sectional study

**DOI:** 10.1265/ehpm.22-00049

**Published:** 2022-06-18

**Authors:** Takafumi Abe, Jun Kitayuguchi, Noritoshi Fukushima, Masamitsu Kamada, Shinpei Okada, Kenji Ueta, Chiaki Tanaka, Yoshiteru Mutoh

**Affiliations:** 1 Center for Community-Based Healthcare Research and Education (CoHRE), Shimane University, Shimane, Japan; 2 Physical Education and Medicine Research Center UNNAN, Shimane, Japan; 3 Department of Preventive Medicine and Public Health, Tokyo Medical University, Tokyo, Japan; 4 Department of Health Education and Health Sociology, School of Public Health, Graduate School of Medicine, The University of Tokyo, Tokyo, Japan; 5 Physical Education and Medicine Research Foundation, Nagano, Japan; 6 College of Sport and Health Science, Ritsumeikan University, Shiga, Japan; 7 Department of Human Nutrition, Tokyo Kasei Gakuin University, Tokyo, Japan; 8 The Research Institute of Health Rehabilitation of Tokyo, Tokyo, Japan

**Keywords:** Preschoolers, Japan, COVID-19, Physical fitness, Fundamental movement skills

## Abstract

**Background:**

Physical inactivity during the coronavirus disease 2019 (COVID-19) pandemic may have hindered the development of fundamental movement skills in preschoolers. This serial cross-sectional study compared fundamental movement skills by age group before and during the COVID-19 pandemic (2019–2020), among Japanese preschoolers aged 3–5 years.

**Methods:**

Of the 22 preschools within Unnan City, Shimane Prefecture, Japan, 21 (95.5%) and 17 (77.3%) participated in the 2019 and 2020 surveys, respectively. We analyzed 608 and 517 preschoolers in both surveys. Fundamental movement skills were objectively assessed with a 25 m run, standing long jump, and softball throw, based on the Japanese physical activity guidelines for preschoolers. Mann–Whitney U tests were used to compare the fundamental movement skills data between periods.

**Results:**

For the 25 m run, participants aged 5 years were faster before than during the pandemic (p = 0.018), while participants aged 3 and 4 years showed no significant differences. Participants aged 3–5 years showed no significant differences before and during the pandemic for the standing long jump (p ≥ 0.072). For the softball throw, all grades scored higher before than during the pandemic (p < 0.001).

**Conclusions:**

These findings suggest that the COVID-19 pandemic impeded the development of fundamental motor skills, especially for object control skills. This highlights the need for interventions aimed at developing fundamental motor skills in preschoolers during and after the pandemic.

**Supplementary information:**

The online version contains supplementary material available at https://doi.org/10.1265/ehpm.22-00049.

## Introduction

The World Health Organization declared the coronavirus disease 2019 (COVID-19) outbreak a pandemic on January 30, 2020 [1]. Subsequently, owing to the rapid spread of the disease, the Japanese government declared a nationwide state of emergency from April 16 to May 14, 2020. The pandemic has secondarily affected young children. For example, objectively assessed physical activity decreased and sedentary time increased [2]. This is a critical problem, as physical inactivity in early childhood may ultimately diminish physical proficiency [3, 4].

Notably, fundamental movement skills (FMS) are developed during the preschool years [5]. Sufficient FMS are associated with appropriate body weight and better cardiorespiratory fitness [6]. In turn, young children with sufficient FMS may participate in high-level physical activity in adolescence [7–9]. Despite the importance of mastering FMS during early childhood, the COVID-19 restrictions have delayed the achievement of minimum proficiency. Two studies have demonstrated the pandemic’s negative effect on the motor development of schoolchildren [10, 11]. However, to the best of our knowledge, no studies have reported the influence of the pandemic on FMS among preschoolers (aged 5 years or younger). Therefore, this study aimed to analyze the differences in FMS by age group before and during the pandemic (2019–2020) in children aged 3–5 years in Unnan City, Shimane Prefecture, Japan.

## Methods

The study was conducted in Unnan City (population 39,032; area 553.4 km^2^), a rural municipality in Shimane Prefecture, Japan. Data for this serial cross-sectional study were obtained from FMS surveys among preschoolers aged 3–5 years (grades 1–3) as part of the Unnan City Early Childhood Exercise Program, based on the Unnan City Basic Educational Plan, developed by the Board of Education and Child Policy Division (BECPD) at Unnan City Hall [12]. All 22 local preschools were invited to participate. In this study, the term preschool includes nursery schools, kindergartens, and authorized centers for early childhood education and care (ECECs). The principal of each preschool decided whether or not to participate. The BECPD issued letters explaining the study to the parents or guardians of all preschoolers. The surveys were subsequently conducted at each preschool from October to November in 2019 and 2020. According to the opt-out process, the parents/guardians were free to ask questions or refuse participation, as explained on the affiliated website. The BECPD approved the collected data for use in secondary research. Personal identifiers were stripped from the data analyzed in this study. The study protocol was approved by the Ethics Committee of Shimane University (#2856).

The Early Childhood Exercise Guidelines in Japan acted as the standardized assessment method according to which FMS were evaluated. These methods included the 25 m run (m/s), standing long jumps (cm) (locomotor skills), and softball throws (m) (object control skills) [13]. Two trials were conducted for each test item, and the best score was analyzed. Anthropometric measurements included objectively measured height (cm) and weight (kg) status without shoes and with light clothing, to the nearest 0.1 cm and 0.1 kg, respectively. The Kaup index [weight in kg/(height in m)^2^] data were divided into two categories employing the following cut-off scores: non-overweight: <18.0; overweight: ≥18.0 kg/m^2^ [14].

The sample’s sex distribution and preschool types were compared before and during the pandemic. We presented continuous anthropometric parameters and FMS data as medians and interquartile ranges, with between-period comparisons by age group conducted via the Mann-Whitney U test. Independent-samples tests were conducted based on the null hypothesis that the participants’ FMS scores by age group are at the same level at the two-time points (see Additional file 1). The percentage of overweight participants in the two surveys was compared using a Chi-squared or Fisher’s exact test. To adjust for multiple comparisons (3 groups × 3 measures = 9 comparisons) in FMS, Bonferroni-adjusted p-values were calculated. Statistical significance was set at *p* < 0.05 for two-sided tests. All the data were analyzed with SPSS version 25.0 (IBM Corp., Armonk, NY, USA).

## Results

Table 1 shows the participants’ characteristics. Of the 22 preschools, 21 (95.5%) and 17 (77.3%) participated in the 2019 and 2020 surveys, respectively. This study included 641 and 539 preschoolers in each survey. After excluding missing data, we analyzed 608 (94.9%) and 517 (95.1%) participants, respectively. The percentage of boys and girls before and during the pandemic was not significantly different in any grade (*p* ≥ 0.055). Further, no significant difference was found before and during the pandemic in the percentage of preschool types (*p* = 0.746).

**Table 1:** Participants’ characteristics

	**Before (2019)**		**During (2020)**		**P value^a^**
	
	**n**	**%**	**n**	**%**	
Overall	608		517		
Boys	318	52.3	251	48.5	0.231
Girls	290	47.7	266	51.5	
Three years old	179		140		
Boys	97	54.2	60	42.9	0.055
Girls	82	45.8	80	57.1	
Four years old	169		171		
Boys	84	49.7	89	52.0	0.745
Girls	85	50.3	82	48.0	
Five years old	260		206		
Boys	137	52.7	102	49.5	0.515
Girls	123	47.3	104	50.5	

Type of preschool	21		17		
Nursery school	10	47.6	10	58.8	0.746
Kindergarten	4	19.0	2	11.8	
ECEC	7	33.3	5	29.4	

ECEC; authorized center for early childhood education and care

^a^P for Chi-square test.

Table 2 shows the comparison of FMS before and during the COVID-19 pandemic. The comparison of anthropometric parameters and FMS before and during the pandemic revealed the following: regarding height, participants aged 3 years were notably taller during the pandemic than before (*p* = 0.005), while those aged 4 and 5 years did not show significant differences (*p* ≥ 0.159). Regarding weight, children aged 3 years were notably heavier during the pandemic than before (*p* = 0.005), while those aged 4 and 5 years again did not indicate a significant difference (*p* ≥ 0.136). There was no significant difference before and during the pandemic for the Kaup index and overweight participants (*p* > 0.09).

**Table 2:** Comparison of fundamental movement skills before and during the COVID-19 pandemic

	**Three years old (first grade)**						**Four years old (second grade)**						**Five years old (third grade)**					
		
	**Before (2019)**		**During (2020)**		**Effect size ** **(r or φ)**	**P ** **value^a^**	**Before (2019)**		**During (2020)**		**Effect size ** **(r or φ)**	**P ** **value^a^**	**Before (2019)**		**During (2020)**		**Effect size ** **(r or φ)**	**P ** **value^a^**
					
	**Median or %**	**(IQR or 95%CI)**	**Median or %**	**(IQR or 95%CI)**			**Median or %**	**(IQR or 95%CI)**	**Median or %**	**(IQR or 95%CI)**			**Median or %**	**(IQR or 95%CI)**	**Median or %**	**(IQR or 95%CI)**		
Anthropometric parameter																		
Height (cm)	97.8	(95.1–100.4)	99.3	(95.3–102.2)	0.16	0.005	105.5	(102.8–107.9)	105.1	(102.0–107.8)	0.08	0.159	111.9	(108.7–115.4)	112	(109.6–115.0)	0.01	0.799
Weight (kg)	14.6	(13.3–15.8)	15.2	(14.0–16.4)	0.16	0.005	16.7	(15.7–18.0)	16.5	(15.0–18.0)	0.08	0.136	18.7	(17.3–20.5)	18.9	(17.5–20.8)	0.05	0.322
Kaup index (kg/m^2^)	15.2	(14.6–15.9)	15.4	(14.9–16.0)	0.08	0.129	15.1	(14.3–15.8)	15.0	(14.4–15.7)	0.02	0.740	14.9	(14.2–15.8)	15.1	(14.4–15.9)	0.07	0.147
Overweight (%)^b^	0.6	(0.0–3.1)	3.6	(1.2–8.1)	0.11	0.090	1.8	(0.4–5.1)	1.8	(0.4–5.0)	−0.001	1.000	5.0	(2.7–8.4)	5.8	(3.0–10.0)	0.02	0.695

Fundamental movement skill																		
25 m run (m/s)	3.4	(3.1–3.6)	3.2	(2.9–3.6)	0.14	0.135	3.9	(3.7–4.2)	3.9	(3.6–4.2)	0.05	1.000	4.3	(4.0–4.5)	4.2	(4.0–4.5)	0.14	0.018
Standing long jump (cm)	77.0	(66.0–88.5)	77.5	(60.0–90.0)	0.01	1.000	99.0	(91.0–112.0)	101.0	(88.0–112.0)	0.01	1.000	113.5	(101.0–125.5)	118.0	(106.0–128.0)	0.12	0.072
Softball throw (m)	3.0	(2.5–3.5)	2.5	(1.5–3.0)	0.28	<0.001	5.0	(3.5–6.0)	4.0	(3.0–5.0)	0.24	<0.001	6.3	(4.5–8.3)	5.0	(4.0–7.0)	0.22	<0.001

IQR; interquartile range, CI; confidence interval

^a^P values for Mann–Whitney U test for continuous data and a Chi-squared or Fisher exact test for categorical data. To adjust for multiple comparisons (3 groups × 3 measures = 9 comparisons) in fundamental movement skills, Bonferroni-adjusted p-values were calculated.

^b^Overweight was dichotomized using a cut-off (≥18.0 kg/m^2^) on the Kaup index [14].

For the 25 m runs, participants aged 5 years were faster before than during the pandemic (*p* = 0.018), while participants aged 3 and 4 years showed no significant differences. All participants aged 3–5 years showed no significant differences in the long jumps before and during the pandemic (*p* ≥ 0.072). For the softball throws, all grades scored higher before than during the pandemic (*p* < 0.001).

## Discussion

In this study, we compared FMS scores in preschoolers before and during the pandemic by age group. We found that all participants scored significantly worse on softball throws during the pandemic, while participants aged 5 had slower 25 m run times during the pandemic. To the best of our knowledge, this is the first study to investigate FMS in preschoolers in the COVID-19 context. However, although the FMS scores worsened during the pandemic, the effect size was small (r ≤ 0.28). One of the reasons might be that the assessment period of this study was short-term (one year). Additionally, possibly because the preschoolers engaged in some physical activity with parents at home during the pandemic. Our findings supported the pandemic’s negative effect on the motor development of schoolchildren as demonstrated in two previous studies in Japan and Portugal [10, 11]. Considering these results, future studies should implement closer monitoring of FMS changes in school children.

Our results indicated participants’ poor performance on the 25 m run and softball throw during the pandemic. Recent reviews support the associations between physical activity level and FMS [3, 15]. Although we did not assess physical activity levels, fewer opportunities for vigorous physical activity during the pandemic may have hindered the development of FMS. Under the emergency conditions, all kindergartens were temporarily closed (from April 18 to May 17, 2020). During this time, the BECPD requested that nursery schools and ECECs students refrain from visiting the childcare facility. However, we were unable to identify whether specific students in these nursery schools and ECECs were absent. During the one-month self-restraint period, home play and preschool activities may not have entailed vigorous physical activity; that is, there were limited opportunities to develop FMS.

Moreover, the participants’ object control skills were poorer than their locomotor skills during the pandemic. Locomotor skills are viewed as phylogenetic because they develop “naturally” and require minimal formal instruction and feedback [16]. By contrast, object control skills are considered ontogenetic because they are more culturally determined and require formal practice and feedback to reach a competent level [16]. Although locomotor skills require adequate practice and instruction to reach proficient performance, the throwing motion may be more affected by personal inexperience rather than running and jumping motions. For example, ball sports typically require specialized conditions (e.g., specialized equipment or a team of participants). The coronavirus may be transmitted by coming into physical contact with objects previously touched by infected individuals [17]. Therefore, preschools may have restricted ball-related activities. However, parents, caregivers, and teachers could incorporate physical activity including movement skill-related components into children’s daily routine while adhering to infection prevention regulations [17–19]. Our study demonstrates the importance of vigorous physical activities in increasing FMS development among preschoolers during the pandemic [20].

Several limitations of this study should be noted. Our findings cannot be generalized because the study was performed in a single rural city. Next, there might have been a selection bias because of 5 non-participating preschools in the 2020 survey. Our results might have underestimated the negative impact of the pandemic on FMS, if these preschools were reluctant to allow children to participate in physical activity during the pandemic. Finally, we did not investigate the potential effects of frequency or type of physical activity in the preschool/home. Our research design also precludes causal inferences.

## Conclusions

Lower performance was exhibited in the 25 m runs and softball throws in preschoolers aged 3–5 years during the pandemic compared to the pre-pandemic statistics. This indicates that FMS growth may have been impeded due to the pandemic restrictions. This highlights the need for interventions aimed at developing FMS in preschoolers during and after the pandemic.

## Abbreviations

COVID-19: coronavirus disease 2019

## Supplementary information

**Additional file 1:** The study methodology.

## Declarations

### Availability of data and material

Data cannot be shared for ethical reasons.
