# Supplementary material for: Fundamental movement skills in preschoolers before and during the COVID-19 pandemic in Japan: a serial cross-sectional study
Source: Environ Health Prev Med. 2022 Jun 18;27:26. doi: 10.1265/ehpm.22-00049 (PMC9283913; doi:10.1265/ehpm.22-00049)
Supplement: Supplementary file 1 — Additional file 1: The study methodology. [file ehpm-27-026-s001.pptx]

## Slide 1
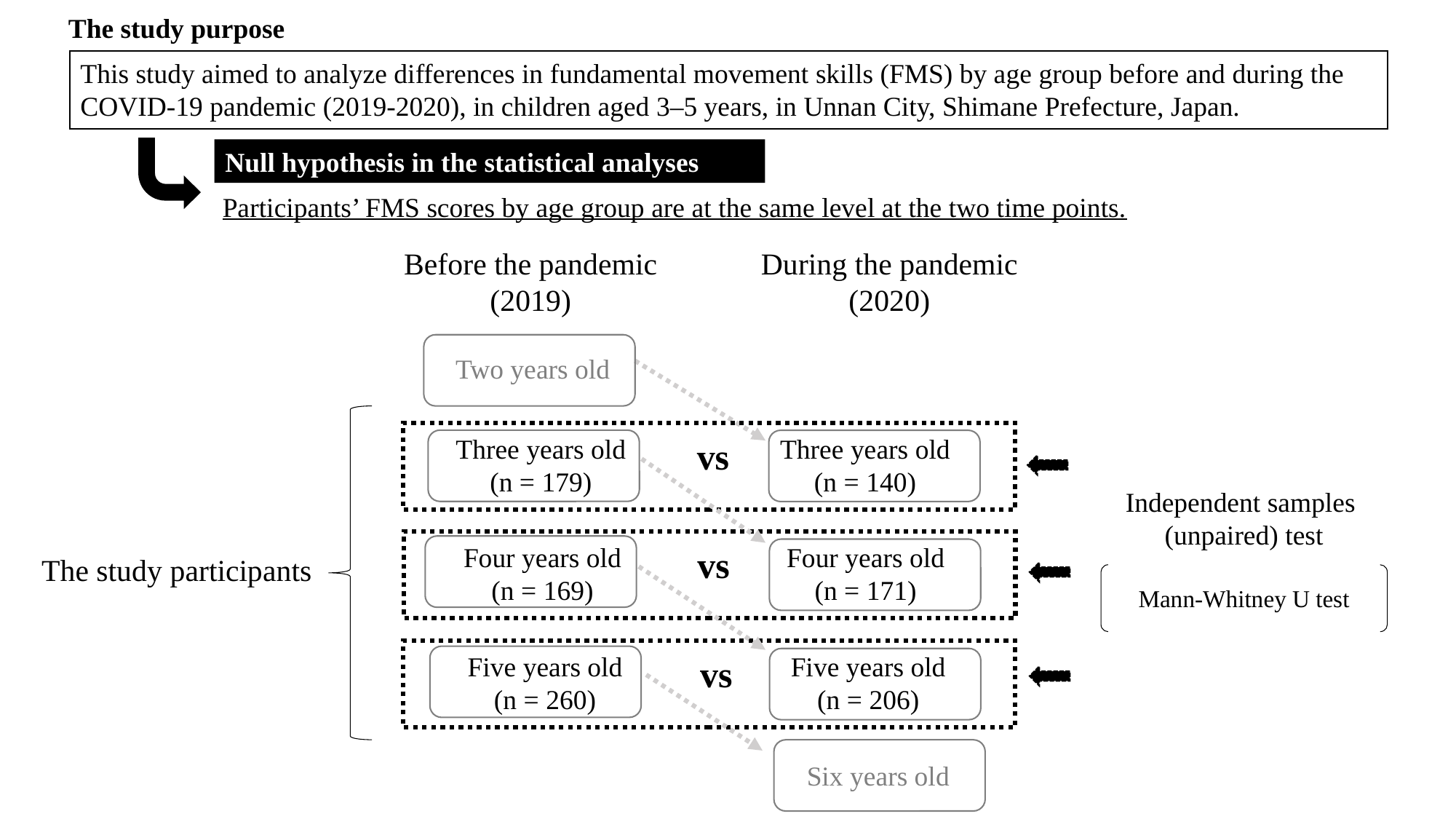

The study purpose
This study aimed to analyze differences in fundamental movement skills (FMS) by age group before and during the COVID-19 pandemic (2019-2020), in children aged 3–5 years, in Unnan City, Shimane Prefecture, Japan.
Null hypothesis in the statistical analyses
Participants’ FMS scores by age group are at the same level at the two time points.
Before the pandemic
(2019)
During the pandemic
(2020)
Two years old
Three years old
(n = 179)
Three years old
(n = 140)
vs
Independent samples
(unpaired) test
Four years old
(n = 169)
Four years old
(n = 171)
vs
The study participants
Mann-Whitney U test
Five years old
(n = 260)
Five years old
(n = 206)
vs
Six years old
